# Is the unruptured intracranial aneurysm treatment score (UIATS) sensitive enough to detect aneurysms at risk of rupture?

**DOI:** 10.1007/s10143-020-01246-x

**Published:** 2020-03-12

**Authors:** Silvia Hernández-Durán, Dorothee Mielke, Veit Rohde, Vesna Malinova

**Affiliations:** Department of Neurological Surgery, Göttingen University Hospital, Robert-Koch-Str. 40, 37075 Göttingen, Germany

**Keywords:** UIATS, PHASES score, Intracranial aneurysm, Subarachnoid hemorrhage

## Abstract

To evaluate if the unruptured intracranial aneurysm treatment score (UIATS) is a sensitive tool to detect aneurysms at risk of rupture, we conducted an a posteriori retrospective study on ruptured intracranial aneurysms. We performed a retrospective analysis of adult patients admitted to our center from January 2010 to April 2016 with aneurysmal subarachnoid hemorrhage. The UIATS was applied to all ruptured aneurysms. Patients for whom the UIATS recommended treatment were labeled as “true positives,” whereas patients for whom the UIATS recommended observation were labeled as “false negatives.” Patients for whom the UIATS was inconclusive were excluded from the final analysis. Based on the UIATS recommendation, a sensitivity analysis was performed. A total of 262 patients with aneurysmal subarachnoid hemorrhage were screened. Of these, 212 were included in our analysis. Median age was 53 years (23–90). Most patients were females (*n* = 134, 63%), with an equal distribution between low-grade and high-grade hemorrhages (Hunt & Hess ≥ 3 *n* = 107, 50%). UIATS recommended treatment in *n* = 52, 25% cases (TP), was inconclusive in *n* = 93, 44% (excluded), and recommended observation in *n* = 67, 32% (FN). Based on these data, the UIATS showed a sensitivity of 44% (CI 35–53%). The UIATS exhibits rather low sensitivity for detecting aneurysms at risk of rupture.

## Introduction

Unruptured intracranial aneurysms (UIA) have an approximate prevalence of 3% worldwide [31], and their management constitutes a great challenge for cerebrovascular specialists. The most feared complication of UIA is aneurysmal subarachnoid hemorrhage (aSAH), which is associated with up to 43% risk of death immediately after ictus, and 57% at 6 months [2, 15]. However, the natural history of UIA remains unclear, since no prospective cohort studies have been conducted to elucidate which one will rupture, and when [25]. The development of such studies has been deemed as unethical, for lack of treatment of UIA could result in a devastating aSAH. Therefore, knowledge about UIA derives from observational studies [17, 33].

The most relevant studies to this matter are the International Study of Unruptured Intracranial Aneurysms (ISUIA) [29] from North American and Europe, with a retrospective arm encompassing 1449 patients with 1937 UIA, and a prospective one with 4060 patients; the unruptured cerebral aneurysm study (UCAS) [11], with 5720 prospectively analyzed Japanese patients; and the study performed by Juvela et al. [13] in 142 patients harboring 181 untreated UIA between 1956 and 1978 in Finland. Based on these works, several risk factors for aneurysm rupture have been identified, such as aneurysm size and location, arterial hypertension, and smoking status.

Taking into consideration these risk factors, clinicians have performed prophylactic treatment of UIA in order to avoid the devastating consequences of aSAH, in spite of the risks associated to therapy and the lack of evidence for a clinical benefit [23]. A meta-analysis of 71 studies revealed 2% mortality at 1 month, and 4.8% of procedural unfavorable outcome in aneurysms treated endovascularly [20]. On the other hand, a meta-analysis on endovascular coiling vs. surgical clipping for UIA revealed 1% mortality and 8–9% ischemia for both treatment modalities [26].

The unruptured intracranial aneurysm treatment score (UIATS) [5] was published in April 2015 as an attempt to summarize risk factors currently considered to play a key role in aneurysm rupture and those related to treatment. The main objective of the authors was to provide clinicians with a tool to guide their decision-making (treatment vs. observation) when dealing with patients harboring UIA based on an international expert consensus. Ultimately, the main goal in the management of UIA is prevention of aSAH without submitting patients to unnecessary treatment risks. Therefore, UIA scoring systems should correctly identify patients harboring UIA in whom the risk of rupture surpasses the treatment risk. Does the UIATS achieve this goal? In this study, we attempt to answer this question.

## Methods

In order to assess whether the UIATS is a sensitive enough tool to identify aneurysms at risk of rupture, we evaluated a cohort of patients with aSAH with the UIATS a posteriori*.*

We performed a retrospective analysis of consecutive adult patients admitted to our center from January 2009 to April 2016 with aSAH. Inclusion criteria comprised (a) age over 18 years and (b) aSAH caused by ruptured saccular aneurysms of the anterior or posterior circulation, confirmed by computed tomography angiography (CT-A) and/or digital subtraction angiography (DSA). Patients harboring mycotic, traumatic, fusiform, or dissecting aneurysms were excluded. Further exclusion criteria were insufficient data on patient files to assess the risk factors comprised in the UIATS.

The UIATS was applied to all ruptured aneurysms (RA). In patients with multiple aneurysms, the lesion considered to be responsible for the aSAH was the only one evaluated by means of UIATS. The risk factors considered in the UIATS are summarized in Table 1. Patients for whom the UIATS recommended treatment were labeled as “true positives (TP),” whereas patients for whom the UIATS recommended observation were labeled as “false negatives (FN).” Patients for whom the UIATS was inconclusive (cumulative scores between − 2 and 2) were excluded from the final analysis. For the purposes of this study, the entire patient cohort was considered as “diseased.”

**Table 1:** Risk factors considered in the UIATS

Factors favoring treatment	Factors favoring observation
Patient age	Life expectancy
Previous SAH from a different aneurysm	Neurocognitive disorder
Familial intracranial aneurysms or SAH	Coagulopathies, thrombophilic disease
Japanese, Finnish, Inuit ethnicity	Psychiatric disorder
Current cigarette smoking	Patient age-related risk
Hypertension (systolic BP > 140 mmHg)	Aneurysm size-related risk
Autosomal polycystic kidney disease	Aneurysm complexity-related risk
Current drug abuse (cocaine, amphetamine)	Intervention-related risk
Current alcohol abuse	
Cranial nerve deficit	
Clinical or radiological mass effect	
Thromboembolic events from the aneurysm	
Epilepsy	
Reduced quality of life due to fear of rupture	
Aneurysm multiplicity	
Aneurysm maximum diameter	
Aneurysm location	
Aneurysm growth on serial imaging	
Aneurysm de novo formation on serial imaging	
Contralateral stenoocclusive vessel disease	

Adapted from [13]

Based on the UIATS recommendation, a sensitivity test was performed. Sensitivity was defined as the ability of the UIATS to identify aneurysms at risk of rupture by recommending treatment in a cohort with proven aSAH. To estimate it, the following equation was employed: sensitivity = TP/(TP + FN).

## Results

A total of 262 patients with SAH were screened. Of these, 212 were included in our analysis. Of the 50 patients excluded, one had a flow-associated aneurysm in the setting of an arteriovenous malformation, one had insufficient data, and 48 had either dissecting or fusiform aneurysms. Mean age was 53 years (23–90). Most patients were females (*n* = 134, 63%), with an equal distribution between low-grade and high-grade hemorrhages (Hunt & Hess ≥ 3, *n* = 107, 50%).

The most common patient-related risk factor observed in our cohort was pre-existing arterial hypertension (*n* = 92, 43%). No patients were of Japanese, Finnish, or Inuit origin. Furthermore, only one patient had a medical history of adult polycystic kidney disease. Illicit drug abuse were present in a minority of patients: active cigarette smoking was observed in *n* = 31, 15%, while current alcohol or drug abuse was reported in *n* = 14, 7%, and *n* = 3, 1%, respectively. Aneurysm multiplicity was seen in *n* = 67, 32%. Most patients had a life expectancy > 10 years before aSAH (*n* = 177, 84%). For a detailed summary of patient-related risk factors, see Table 2.

**Table 2:** Patient-related risk factors considered by UIATS

		*N*	%
Age	< 40 years	29	13.7
	40–60 years	120	56.6
	61–70 years	31	14.6
	71–80 years	20	9.4
	> 80 years	12	5.7
Risk factor incidence	Previous SAH from a different aneurysm	0	0
	Familial intracranial aneurysms or SAH	1	0.5
	Japanese, Finnish, Inuit ethnicity	0	0
	Current cigarette smoking	31	14.6
	Hypertension (systolic BP > 140 mmHg)	92	43.4
	Autosomal polycystic kidney disease	1	0.5
	Current drug abuse (cocaine, amphetamine)	3	1.4
	Current alcohol abuse	14	6.6
Clinical symptoms related to UIA	Cranial nerve deficit	0	0
	Clinical or radiological mass effect	3	1.4
	Thromboembolic events from the aneurysm	1	0.5
	Epilepsy	1	0.5
Other	Reduced quality of life due to fear of rupture	0	0
	Aneurysm multiplicity	67	31.6
Life expectancy due to chronic and/or malignant diseases	< 5 years	19	8.9
	5–10 years	16	7.5
	> 10 years	177	83.5
Comorbid disease	Neurocognitive disorder	2	0.9
	Coagulopathies, thrombophilic diseases	2	0.9
	Psychiatric disorder	10	4.7

Aneurysms were predominantly located in the anterior communicating artery (ACOM) (*n* = 75, 35%), followed by the middle cerebral artery (MCA) (*n* = 48, 23%). Aneurysms in the posterior circulation were responsible for a minority of aSAH (*n* = 33, 16%). The most frequent aneurysm-related risk factors observed in our cohort were irregularity or lobulation (*n* = 108, 51%), and aspect ratio > 1.6 or size ratio > 3 (*n* = 51, 46%). Interestingly, aneurysms were rather small: most of them were between 4.0 and 6.9 mm in diameter (*n* = 86, 40%), followed by those between 7.0 and 12.9 mm (*n* = 81, 38%), and those < 3.9 mm (*n* = 28, 13%). Further aneurysm-related risk factors are summarized in Table 3.

**Table 3:** Aneurysm-related risk factors considered by UIATS

		*N*	%
Maximum diameter	≤ 3.9 mm	28	13.2
	4.0–6.9 mm	86	40.6
	7.0–12.9 mm	81	38.2
	13.0–24.9 mm	15	7.1
	≥ 25 mm	2	0.9
Morphology	Irregularity or lobulation	108	50.9
	Size ratio > 3 or aspect ratio > 1.6	118	55.7
Location	Basilar bifurcation	20	9.4
	Vertebral/basilar artery	4	1.9
	Anterior communicating artery or posterior communicating artery	94	44.3
Other	Aneurysm growth on serial imaging	0	0
	Aneurysm de novo formation on serial imaging	1	0.5
	Contralateral stenoocclusive vessel disease	13	6.1

**Fig. 1:**
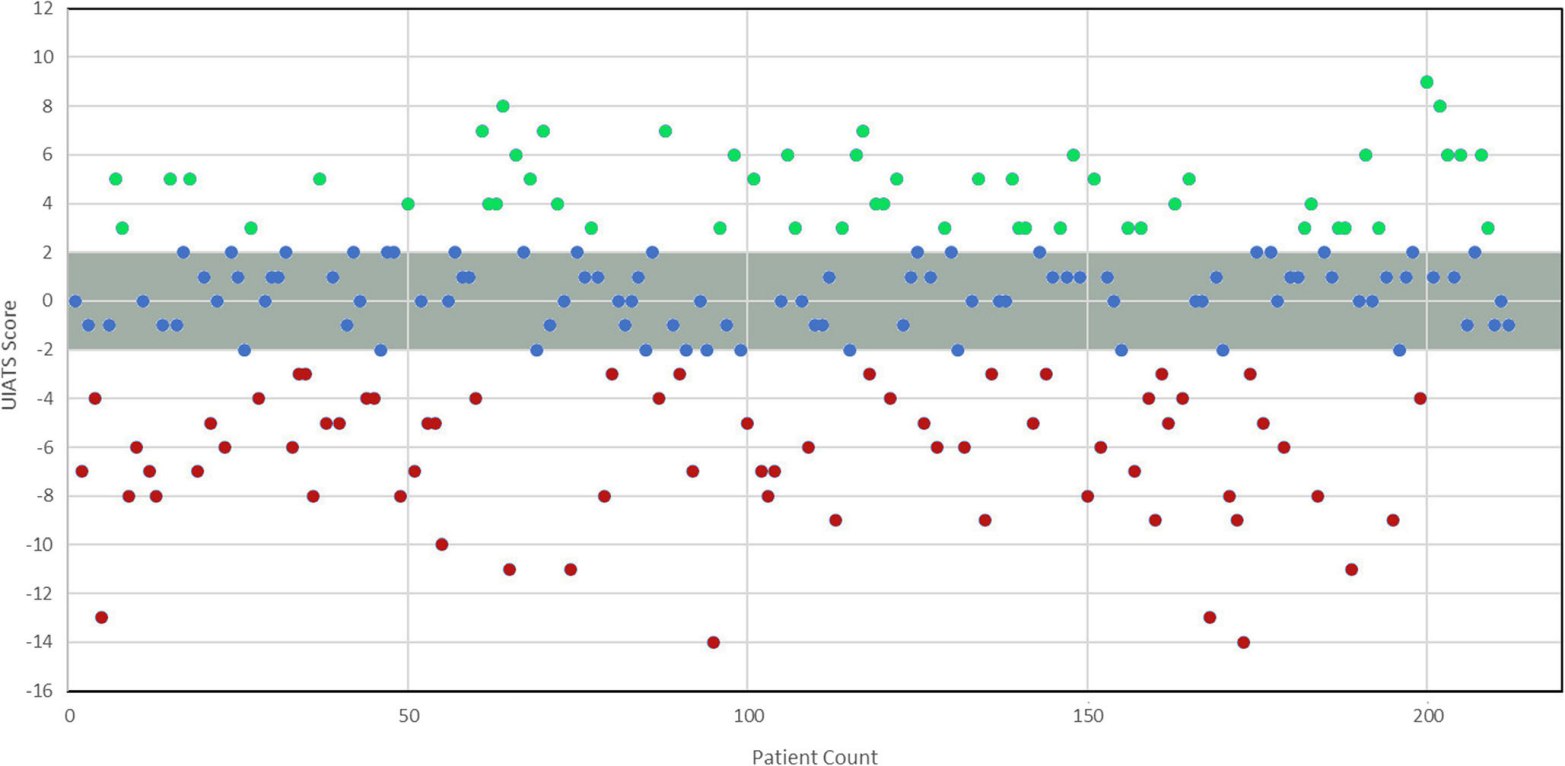
UIATS results in the present cohort. The y-axis denotes the cumulative UIATS: red dots denote scores favoring observation, while the green dots denote scores favoring treatment. The blue dots highlighted in the gray area are the cases in which the UIATS was inconclusive

UIATS recommended treatment in *n* = 52, 25% cases (TP) was inconclusive in *n* = 93, 44% (excluded), and recommended observation in *n* = 67, 32% (FN). The distribution of the UIATS results is summarized in Fig. 1. Based on these data and as illustrated in the contingency table (Table 4), the UIATS showed a sensitivity of 44% (CI 35–53%).

**Table 4:** Contingency table for the UIATS

	Disease present (ruptured aneurysm)	Disease absent (unruptured aneurysm)
UIATS in favor of treatment	52 (true positive)	0 (false positive)
UIATS in favor of observation	67 (false negative)	0 (false negative)
Sensitivity: 52/(52 + 67) = 44%		

## Discussion

One of the most complex questions currently faced by cerebrovascular surgeons is which UIA should be treated. To date, there are no unequivocal criteria by which to estimate the risk of rupture of a given UIA, since several factors can interact and determine the natural history of a lesion.

In 2014, Greving et al. [7] published the PHASES score as an aid for prediction of the risk of rupture of UIA. Based on a review and pooled analysis of individual patient data from 8382 participants in six prospective trials [11, 13, 18, 28, 29, 32], the authors identified age, hypertension, history of SAH, aneurysm size, aneurysm location, and geographical region as predictors for UIA rupture. The sum of these factors then yields a 5-year rupture risk, which can guide clinicians and patients in their decision-making on whether or not a UIA should be treated.

Bijlenga et al. [1] recently published a population-based study in which they assessed whether the PHASES score would aid in identifying patients with UIA at low risk of rupture. They prospectively included 841 patients in their analysis. They were then stratified into four groups: (a) patients with stable UIA; (b) patients with growing UIA; (c) patients with treated UIA; and (d) patients with aSAH. The authors found that the odds of being diagnosed with an aSAH were associated with PHASES score > 3. In turn, a PHASES score of ≤ 3 was associated with a low likelihood of aneurysm rupture, thus validating this tool for estimation of UIA rupture risk.

However, the PHASES score has undergone heavy criticism because certain groups of patients were underrepresented. For example, smoking [3, 12, 27] and positive familial history of aSAH [14, 16, 21], well-established risk factors for UIA rupture, were not considered in the score. Furthermore, this score is based on pooled data from observational studies, where aneurysm treatment was left to physician’s discretion, thus rendering it subject to attrition bias. In their letter to the editor, Darsaut et al. [4] even go as far as equating the PHASES score to pseudoscience.

In a recent publication by Hilditch et al. [9], the PHASES score was evaluated in similar manner to our study in a cohort of RA. Among the 700 patients included, 17% had a PHASES score of 3 or less. The authors conclude that a considerable number of patients would have been managed conservatively based on their PHASES score, had they presented incidentally prior to rupture. Consequently, the PHASES score alone should not dictate treatment decisions, since it may erroneously classify patients as low risk for rupture.

The UIATS was developed shortly after the PHASES score as a multidisciplinary consensus model, in which patient-related, aneurysm-related, and treatment-related risk factors were considered in quantitative fashion. Unlike the PHASES score, the UIATS took into consideration treatment-related risk factors, as well as aneurysm-related and patient-related risk factors, thus providing a more comprehensive assessment of all the variables that might influence treatment decision-making in UIA. Nevertheless, its aim was not to provide a prognostic nor a predictive model for UIA rupture, but a reflection of contemporary practice in UIA management and guidance to clinicians treating patients with UIA.

In October 2017, Ravindra et al. [22] published a single-center validation study for the UIATS. Here, the authors scored 221 patients with UIA according to the UIATS. Subsequently, they were categorized in a contingency table assessing the UIATS recommendation versus real-world treatment decision. Percentage of misclassification, sensitivity, specificity, and area under the receiver operating characteristic (ROC) curve were calculated. In this study, the UIATS recommendation was significantly associated with the actual treatment of UIA, while sensitivity and specificity were 49% and 80%, respectively. One of the main points of criticism to this study is the calculation of sensitivity and specificity of the UIATS based on real-world treatment decisions within the authors’ quaternary academic medical center and the UIATS recommendation. The former assumes that those treatment decisions were correct; it presupposes that no over- or undertreatment took place in their center, and that their practice equated a sort of “gold standard.”

Similarly, our group published in February 2018 [8] an evaluation study of the UIATS, in which we attempted to elucidate whether the UIATS reflects daily clinical practice in a cerebrovascular reference center. We evaluated 93 patients harboring 147 UIA. A positive correlation coefficient of 0.366 in Spearman’s rank order was observed, thus showing accordance between our more intuitive treatment decisions and the UIATS.

The present study is the first one to assess the UIATS’ ability to identify intracranial aneurysms at risk of rupture a posteriori*.* While the methodology of our study was rather simple, it provides understandable data in regard to a straightforward question: could the UIATS correctly identify aneurysms at risk of rupture? Our study suggests that the UIATS does not have sufficient sensitivity to be considered a reliable tool in UIA decision-making. In almost half of our patient cohort, the UIATS failed to provide a recommendation (inconclusive cases). Additionally, almost a third of the patients were categorized as FN, since the UIATS recommended observation. Cumulatively, almost 75% of our patient cohort would have been erroneously classified. Had these patients presented incidentally before rupture, they could have succumbed to a fatal aSAH due to misclassification.

Of note, most of the ruptured lesions in our cohort exhibited a rather small size, which might have contributed to a lower UIATS and thus their misclassification as aneurysms amenable to observation. Furthermore, most patients were female, a risk factor for UIA rupture well described in the literature [11, 13, 29] but not considered in the UIATS. Patient age, aneurysm size, and complexity are considered twice in the UIATS: as factors favoring observation and as factors favoring treatment. This can also render the results equivocal. Additionally, the intervention-related risk is scored with a constant in the UIATS. It is well established that physician’s experience correlates with procedure risk; treatment of an aneurysm at a high-volume cerebrovascular center is not equivalent to treatment at a small institution [24].

As noted by Indrayan and Malhotra [10], a scoring system is useful only when it provides new information in regard to a complex condition and quantifies aspects that may be difficult to stratify in daily clinical assessment. Bearing this in mind, the UIATS is only useful in that it comprises complex data considered by experts when making treatment decisions for UIA. Nevertheless, it does not provide any new information.

Fahed et al. [6] offer interesting criticism to the UIATS. The authors argue that the methodology employed to develop the UIATS, namely the Delphi process, was designed for business and warfare forecasting, with little relation to medical sciences. This, in turn, yields a harmonized opinion, but no facts on which to base a treatment decision in the era of evidence-based medicine. To date, scientific papers have attempted to validate both the PHASES and the UIATS in relation to their accordance with current clinical practice, assuming correctness of said practice. However, there is no way to know whether treated UIA would have ruptured and led to aSAH; the only way to know this would be in the framework of a prospective cohort study. As mentioned, such trial would pose an ethical challenge, since observation of UIA could lead to aSAH. Taking into consideration the studies mentioned in this discussion, both PHASES and UIATS offer pooled data and homogenized opinions, but no hard data on which to base treatment decisions regarding UIA.

In the last decade, hemodynamic parameters have emerged as potential markers of rupture risk in UIA. Some of these parameters include wall shear stress (WSS) and oscillatory shear index (OSI), which can be measured by computational fluid dynamics (CFD) [19]. Two of the largest studies in this respect were conducted by Xiang et al. [34] and Zhang et al. [35], including 204 and 173 aneurysms, respectively. In these papers, RA and UIA were analyzed, showing that low WSS and high OSI correlated with rupture risk. Taking into consideration that WSS has been associated with pathobiological changes in the aneurysm wall, such as disruption of the internal elastic lamina, smooth muscle cell migration, inflammatory infiltration, and loss of collagen [30], the use of these parameters in rupture risk prediction seems promising. While CFD is still being developed and refined, the incorporation of this technology can provide objective criteria to guide clinical decision-making and complement current scoring systems, such as PHASES and UIATS.

## Study strengths and limitations

Our study is retrospective in nature with a relatively small patient population. The retrospective design renders the study subject to several biases, in particular, data quality assurance. Risk factors contained in the social history, such as tobacco or alcohol consumption, might not have been accurately documented. This, in turn, might have skewed the UIATS towards observation instead of treatment. A larger, prospective cohort might have yielded different results, possibly in greater accordance with the UIATS.

On the other hand, because of the methodology of our study, predictive values and specificity could not be calculated, as the entire cohort was considered as “diseased.” The former would presuppose a disease prevalence of 100%, which does not reflect the real prevalence of aSAH or UIA. Therefore, the estimation of predictive values and accuracy cannot be conducted in a reliable fashion. While this is also a potential limitation of the study, our results serve to illustrate how fallible the UIATS can be.

## Conclusion

Treatment decision-making in patients with UIA remains difficult and controverted. To date, no prospective cohort studies exist to unequivocally answer the question of which UIA should be treated in order to prevent devastating aSAH. In such a dubious scientific environment, the UIATS provides a quantification of expert-assessed risk factors for UIA rupture. While useful when considering all the variables that should determine UIA treatment, the UIATS is not an empiric, mathematical model. Consequently, the UIATS should be regarded as a harmonized expert opinion on a complex subject matter, not fact. The authors thus recommend the use of UIATS as an adjunct in UIA treatment decision-making, but not as a stand-alone tool.
